# Effect of Foliar Application of Hydrogen Peroxide Macroconcentrations on Growth Parameters, Phenolic Compounds and Antioxidant Capacity in the Leaves and Seeds of *Amaranthus hypochondriacus* L.

**DOI:** 10.3390/plants12071499

**Published:** 2023-03-29

**Authors:** Roberto Augusto Ferriz-Martínez, Nayeli Espinosa-Villarreal, Jorge Luis Chávez-Servín, Adán Mercado-Luna, Karina de la Torre-Carbot, Juan Serrano-Arellano, Carlos Saldaña, Teresa García-Gasca

**Affiliations:** 1 Laboratorio de Biología Celular y Molecular, Facultad de Ciencias Naturales, Campus Juriquilla, Universidad Autónoma de Querétaro, Av. de las Ciencias S/N, Juriquilla, Querétaro 76320, Mexico; 2 Departamento de Biosistemas, Facultad de Ingeniería, Centro de Investigación y Desarrollo Tecnológico en Materia Agrícola, Pecuaria, Acuícola y Forestal (CIDAF), Campus Amazcala, Universidad Autónoma de Querétaro, Carretera a Chichimequillas S/N, Amazcala, El Marqués, Querétaro 76130, Mexico; 3 División de Estudios de Posgrado e Investigación, Tecnológico Nacional de México/IT de Pachuca, Carretera México-Pachuca Km, 87.5, Colonia Venta Prieta, Pachuca de Soto, Hidalgo 42080, Mexico; 4 Laboratorio de Biofísica de Membranas y Nanotecnología, Unidad de Microbiología Básica y Aplicada, Facultad de Ciencias Naturales, Campus Aeropuerto, Universidad Autónoma de Querétaro, Anillo Vial Junípero Serra, Querétaro 76140, Mexico

**Keywords:** amaranth, elicitor, hydrogen peroxide, phenolic compounds, secondary metabolites

## Abstract

Amaranth has many interesting features, both nutritional and otherwise, that make it attractive as a food crop. Plants grown in greenhouses have higher yields but lower nutritional value compared to those grown in open fields. This prompted an interest in studying viable elicitors for the production of amaranth. Small hydrogen peroxide (H_2_O_2_) concentrations for foliar spraying from 0 to 18 mM have been used in greenhouse amaranth cultivation. The objective of this work was to evaluate the effect of foliar application of H_2_O_2_ megadoses on growth parameters, total phenolic compounds, condensed tannins, anthocyanins, and the antioxidant capacity of leaves and seeds of amaranth grown in a greenhouse setting. The seed of the *Amaranthus hypochondriacus* L. species was used. The concentrations of H_2_O_2_ analyzed were 0, 125, 250 and 400 mM, with 11 applications throughout the growing cycle. The variable data were subjected to an analysis of variance (ANOVA), followed by a Tukey’s *post hoc* test (95% CI, *p* < 0.05). The results on chlorophyll, growth parameters and proximal chemical analysis showed no statistical difference between the control group versus the treatment groups. A greater number of favorable changes in the different variables studied were observed with the 125 mM H_2_O_2_ treatment, including the increase in antioxidant capacity measured by FRAP. The seed showed a considerable increase in TFC with all treatments and responded better to the 250 mM H_2_O_2_ treatment in the case of DPPH (an increase of 30%) and TPC (an increase of 44%). A 28% increase in anthocyanin content was observed with the treatment of 400 mM H_2_O_2_. The use of H_2_O_2_ may be an appropriate strategy to enhance the production of antioxidant compounds in amaranth without affecting growth or its basic proximal chemical composition. More studies are required in this regard.

## 1. Introduction

*Amaranthus* has been called the crop of the third millennium. There are about 400 species of the genus *Amaranthus* distributed around the world [1,2]. In Mexico, the four most prevalent amaranth seed-producing species are *A. hypochondriacus*, *A. cruentus*, *A. caudatus* and *A. hybridus* [3]. *Amaranthus hypochondriacus* has good adaptation, a high resistance to drought, and is fast-growing. The whole plant (roots, stems, leaves and seeds) is suitable for human and animal consumption. The consumption of amaranth is popular in many parts of Asia and the Americas for its high nutritional value and its content of dietary fiber, essential amino acids, chlorophyll, vitamin C, phenolic compounds, flavonoids, lycopene, unsaturated fatty acids, glucosinolates, proteins, soluble peptides, squalene and beta-carotene [4,5]. It also has potential industrial applications, for example as a source of bioactive molecules and proteins or for cosmetics, dyes, and biodegradable plastics production [6,7]. *A. hypochondriacus* is the most robust and highest yielding of this group, and it can be grown in the open field as well as in a greenhouse. Greenhouse cultivation in a controlled environment has the advantage of increasing crop yield in comparison to the open field system, but it has at the same time been found to result in lower nutritional quality [lower protein content (−9.0%), lipids (−17.6%), ash (−43.1%) and smaller seed size (−22.5%)] [6]. In recent decades, greenhouse cultivation has become an increasingly prevalent practice for many plants due to the large number of variables that can be controlled within certain limits (temperature, humidity, light intensity, pests, external pollutants, CO_2_ concentration, irrigation, etc.). This can improve production and product yield with less waste, given the necessary infrastructure [8]. However, in improving the quantity of food production, food science and technology must not lose sight of the nutritional quality and the secondary metabolites with bioactive potential that are obtained. Secondary metabolites are natural compounds produced by plants that have a variety of health benefits. In recent decades, they have gained interest in traditional medicine and human health. Among the secondary metabolites produced by plants (terpenes, phenolic compounds, glycosides and alkaloids), phenolic compounds are the most widely studied. They include anthocyanins, flavonoids, tannins, and others. Phenolic compounds have been evaluated for their beneficial effects, including anti-inflammatory, antioxidant, antimicrobial, diabetes mellitus, malaria, hypercholesterolemia, atherosclerosis, helminthic diseases, hepatic diseases, cardiovascular complications and cancer-fighting properties [4,9,10,11]. Various bioactive phenolic compounds, such as flavonoids, are found in the vegetal structure of the plant. They have the ability to inhibit or regulate inflammation, and antioxidant and immunomodulatory properties are also attributed to them, associated with their free radical scavenging activity, metal chelation capabilities, and ability to bind proteins with a high degree of specificity. Phenolic compounds have at least one phenol group, an aromatic ring linked with one or more hydroxyl groups, and their various structures have been associated with beneficial health effects for those who consume them. More than 10,000 structures have been identified [12].

Food plants like amaranth contain varying amounts of components with antioxidant properties. Therefore, the evaluation of the antioxidant capacity in the plant by different techniques allows for a global measurement of the inhibition of reducing molecules [13,14]. Many biotechnological strategies have been applied to improve productivity, but elicitation is recognized as the most feasible for increasing production of secondary metabolites with antioxidant capacity in plants [15]. Elicitation refers to the synthesis of secondary metabolites triggered by the action of factors called elicitors or inducers. These factors can be exogenous (produced by pathogenic organisms, mechanical damage or chemical agents) or endogenous (produced by plants in response to certain stress situations). Reports by Siyuan Jin in 2021 observed that the use of LED 460 nm blue light as an elicitor improved the content of chlorophyll and soluble solids, ascorbic acid and antioxidant capacity in freshly cut amaranth [16]. The elicitor stimulates the defense mechanisms of the plant against biological and non-biological agents and induces phenolic biosynthesis [17,18]. Many types of elicitors of diverse chemical nature have been identified, such as inorganic salts, carbohydrates, oligoglycans, lipids, fatty acids, chitosan-type oligomers, polypeptides, ethylene, algae extracts, methyl jasmonate, ascorbic acid, hydrogen peroxide, and others [19,20,21]. Hydrogen peroxide (H_2_O_2_) has been studied for its capacity to increase bioactive compounds in plants, acting as a signal molecule involved in defense responses triggered in the plant under different stress conditions. Hydrogen peroxide diffuses freely across membranes and is relatively long-lived, playing a central role in stress signal transduction pathways [22]. In a past study, H_2_O_2_ concentrations of 6, 14 and 18 mM were applied to leaves during the cultivation cycle of *A. hypochondriacus*. These low concentrations were observed to be effective in moderately increasing antioxidant compounds [17]. It is not known, however, whether the higher concentrations reported in other studies (150–200 mM) [21,23] might elicit more of an increase in bioactive compounds without detriment to plant growth. The objective of this work was to evaluate the effect of the foliar application of hydrogen peroxide in concentrations of 125, 250, and 400 mM on growth parameters, total phenolic compounds, condensed tannins, anthocyanins and antioxidant capacity of leaves and seeds of *A. hypochondriacus* grown in a greenhouse.

## 2. Results

### 2.1. Proximate Analysis

The results of the proximal chemical analysis (Table 1) consist of the percentages of moisture (leaf: 76.3 ± 2.1%; seed: 9.08 ± 1.2%), crude ash (leaf: 3.6 ± 0.1%; seed: 3.12 ± 0.1%), crude fiber (leaf: 2.6 ± 0.1%; seed: 5.9 ± 0.2%), crude protein (leaf: 3.9 ± 0.2%; seed: 17.08 ± 0.1%), and ether extract (leaf: 0.3 ± 0.1%; seed: 4.30 ± 0.2%). No statistical difference (*p* > 0.05) was found between the control group versus the groups with hydrogen peroxide treatments (0, 125, 250 and 400 H_2_O_2_ mM). Likewise, no statistical difference (*p* > 0.05) was observed in the proximal chemical composition between the different sampling times: 63, 90 and 127 days.

### 2.2. Chlorophyll

Chlorophyll determination was carried out twice during the growing period. No statistical difference was found between the control group versus the H_2_O_2_ treatments. In the first chlorophyll determination (90 days after sowing), SPAD values for the different treatments were: 43.82 ± 6.4 (0 H_2_O_2_ mM), 43.54 ± 4.4 (125 H_2_O_2_ mM), 42.01 ± 5.3 m (250 H_2_O_2_ mM) and 42.70 ± 5.1 (400 H_2_O_2_ mM). In the last chlorophyll measurement (104 days after sowing), SPAD values of 42.55 ± 6.1 (0 H_2_O_2_ mM), 42.00 ± 5.0 (125 H_2_O_2_ mM), 42.20 ± 5.6 (250 H_2_O_2_ mM), and 40.00 ± 6.0 (400 H_2_O_2_ mM) were found. No increase or decrease in this variable was observed over time.

### 2.3. Growth Parameters

**Plant height:** This measurement began 41 days after sowing, when the amaranth samples measured approximately 45 cm, and continued until the harvest, when they reached up to 304 cm. No statistical differences (*p* > 0.05) were found between the control group versus the treatments with 0, 125, 250 and 400 mM hydrogen peroxide (Table 2).

**Stem diameter:** The stem diameter measured more than 1.0 cm at the beginning of the determinations and increased to up to 2.45 cm at the end of the crop cycle. The stem diameter increased throughout the crop cycle. No statistical differences (*p* > 0.05) were found between the control group versus the treatments (Table 2).

**Number of leaves:** Over time, the plants develop a larger foliar area, and the number of leaves increase throughout the plant’s growing cycle. No statistical differences (*p* > 0.05) were found between the control group versus the hydrogen peroxide treatments between the 41st (23 leaves) and 76th (155 leaves) days (Table 2).

**Panicle length:** Panicle length was measured (99 cm) at the time of the amaranth seed harvest (134 days). No statistical differences (*p* > 0.05) were found between the control group versus the treatments (Table 2).

### 2.4. Antioxidant Capacity

**DPPH:** three leaf samples were taken throughout the cultivation period (at 63, 90 and 127 days). In the first (at 63 days), antioxidant capacity in the leaf was found to be the highest in leaves treated with 125 mM H_2_O_2_, with a value of 556.44 mg AAE/100 g DM (IC50 = 24 µg/mL), 18% higher than the antioxidant capacity of the control group (472.50 mg AAE/100 g DM). In contrast, with the 400 mM H_2_O_2_ treatment, antioxidant capacity was 17% lower than in the control group. In the second sampling (at 90 days), antioxidant capacity determined by DPPH was found to decrease with the application of all foliar hydrogen peroxide treatments, most notably in the treatment with 250 and 400 mM H_2_O_2_, which showed a value of 118.86 and 124.93 mg AAE/100 g DM, 59 and 57% lower, respectively, than the control group (288.04 mg AAE/100 g DM). In the last sampling (127 days), a decrease in antioxidant capacity was also observed with all treatments (125, 250 and 400 mM H_2_O_2_), averaging 13% lower than the control group (585.02 mg AAE/100 g DM; 90.2% inhibition percentage (IC50 = 19.9 µg/mL)) (Table 3).

Regarding the seed, antioxidant capacity was 30% higher in plants treated with the 250 mM H_2_O_2_ treatment (32.42 93 mg AAE/100 g DM) than in the control group (24.95 mg AAE/100 g DM) and 33% lower than the control group in plants treated with 125 mM H_2_O_2_ (16.72 mg AAE/100 g DM) (Table 3).

**FRAP:** Antioxidant capacity was also determined by the FRAP method in amaranth leaf and seed (*Amaranthus hypochondriacus* L.). In all three leaf samplings treated with 125 nm H_2_O_2_, antioxidant capacity was found to be higher than in the control group: in the first sampling (at 63 days), it was 22.5% higher (3.26 g AAE/100 g DM). In the second sampling (at 90 days), the antioxidant capacity of the leaf (6.54 g AAE/100 g DM) was observed to be 9% higher than the control group. In the third sampling (at 127 days), a positive difference of 22% (8.57 g AAE/100 g DM) was observed, while increases of 1% and 10% were observed in the other two treatment groups: 250 and 400 mM H_2_O_2_, respectively, against the control group (7 g AAE/100 g DM). In amaranth seed, antioxidant capacity was found to be 10% lower than the control group in the group treated with 400 mM H_2_O_2_ (0.59 AAE/100 g DM). In general, the antioxidant capacity determined by FRAP increased in all the groups at 127 days. In addition, in the three sampling times, the best result was obtained with the 125 mM H_2_O_2_ treatment (Table 3).

### 2.5. Total Phenolic Compounds

Increases in total phenolic compounds were found in plants whose leaves were treated with hydrogen peroxide: in the first sampling (at 63 days), they were found to be 8%, 12% and 25% higher in plants treated with 125, 250 and 400 mM H_2_O_2_, respectively, than the control group (254.40 mg GAE/100 g DM). In the second leaf sampling (at 90 days), the 125 and 250 mM H_2_O_2_ treatment groups showed readings 21% and 30% higher, respectively, than the control group (373.66 mg GAE/100 g DM). In the third sampling (127 days), the reading was 21% higher in the 125 H_2_O_2_ mM treatment group. In the amaranth seed, all three treatment groups (125, 250 and 400 mM H_2_O_2_) showed higher total phenolic compounds (24%, 44% and 12%, respectively), over the control group (130.21 mg GAE/100 g DM) (Table 4).

### 2.6. Condensed Tannins

Condensed tannins were found in higher amounts in all three leaf samplings of plants subject to foliar hydrogen peroxide treatments at 125 mM H_2_O_2_: In the first sampling (at 63 days), amounts were 10% and 41% higher in the 125 mM and 250 mM H_2_O_2_ treatment groups, respectively, than the control group (948.65 mg CE/100 g DM). In the second (at 90 days), the reading for the 125 mM H_2_O_2_ treatment group was 36% higher, but 8.37% and 24% lower for the 250 and 400 mM H_2_O_2_ treatment groups, compared to the control group (1632 mg EC/100 g DM). In the third leaf sampling (at 127 days), all three treatment groups showed higher condensed tannins (16%, 24% and 19% higher, respectively, in the 125, 250 and 400 mM H_2_O_2_ groups, compared to the control group (1274.26 mg EC/100 g DM). In the seed, no statistical differences (*p* > 0.05) were found in the condensed tannin values between the control group versus any of the H_2_O_2_ treatments (Table 5).

### 2.7. Anthocyanins

Anthocyanins were found to be higher in plants subject to hydrogen peroxide foliar treatments in all three leaf samplings: In the first (at 63 days), increases of 31%, 26% and 22% were observed for the 125, 250 and 400 mM H_2_O_2_ treatments, respectively, compared to the control group (667.54 mg CGE/100 g DM). In the second (at 90 days), increases of 6%, 2% and 4% were observed for the 125, 250 and 400 mM H_2_O_2_ treatment groups, respectively, compared to the control group (870.13 mg CGE/100 g DM). In the third (at 127 days), increases of 7%, 2% and 2% were observed for the 125, 250 and 400 mM H_2_O_2_ treatment groups, respectively, compared to the control group (832 mg CGE/100 g DM). Regarding the seed, the anthocyanin content increased (*p* < 0.05) only in the 400 mM H_2_O_2_ treatment group, a difference of 28% compared to the control group (130.65 mg CGE/100 g DM) (Table 6).

## 3. Discussion

In the parameters for plant growth evaluated in the present study, no statistical difference (*p* > 0.05) was found between the control group versus the groups subjected to the hydrogen peroxide treatments used. This indicates that the concentrations and frequency of application did not affect amaranth development in terms of number of leaves and panicle length, stem diameter and plant height throughout the cultivation period in the greenhouse. Likewise, regarding the proximal chemical composition, in all the variables analyzed, no statistical differences were found between the control group versus the groups subject to foliar H_2_O_2_ treatment. There were also no differences in the chlorophyll values. A study that analyzed four varieties of *Amaranthus hypochondriacus* (Gabriela, Tulyehualco, DGETA and Nutrisol) reported differences in the proximal chemical analysis in seed (but not in leaves), finding, compared to our results, a greater quantity of crude fiber 173% and crude protein 9.8% in seeds grown in open fields in arid zones of the Mexican Altiplano [24]. However, for the rest of the parameters studied in the leaf, values were similar to the proximal values of our samples. Likewise, our results are consistent with the values reported in another study that analyzed the proximal chemical composition of *Amaranthus hypochondriacus* leaf and seed [25], although in our study a greater amount of crude fiber 1.5% and crude protein 2.3% was found in the seed. These variations are to be expected in seeds of the same species grown under different agroclimatic conditions and different cropping systems.

In this study, hydrogen peroxide did not affect the plants’ growth parameters or proximal chemical composition. A differential effect was observed, however, in the antioxidant capacity, with significant increases (*p* < 0.05) observed in the content of total phenolic compounds, condensed tannins and anthocyanins. Although a differential effect was observed in the antioxidant capacity determined by DPPH and FRAP in the leaves and seeds of amaranth, no regular pattern was observed between the different concentrations of hydrogen peroxide (125, 250 and 400 mM) in foliar application or between the three leaf sampling times (63, 90 and 127 days). Antioxidant capacity increased only moderately, and with different concentrations of hydrogen peroxide, decreases of up to 59% in antioxidant capacity (measured by DPPH) were observed in the leaf. This means there was no dose-response effect observed and that there may be other associated factors that operate in the defense mechanisms of plants, such as the phenological stage and the botanical structure analyzed (leaves and seed).

Although no regular pattern can easily be determined, it can be observed that with the 125 mM H_2_O_2_ treatment, a greater number of favorable changes in the different variables studied were observed, including an increase in antioxidant capacity measured by FRAP. Additionally, both in the tender leaf (sampling at 63 days) and in the third sampling (day 127), more increases were observed in the concentrations of the metabolites studied. It is important to note that the seed showed a considerable increase in TFC under all treatments and responded better to the 250 mM H_2_O_2_ treatment in the case of DPPH, with an increase of 30%, indicating a considerable increase in antioxidant capacity and a 44% increase in TPC, than to the treatment with 400 mM H_2_O_2_, with a 28% increase in anthocyanin content.

Hydrogen peroxide at low levels plays a role as a signaling molecule for proper growth and development of plants [26,27]. However, further study is needed to determine what concentrations should be used in each crop, depending on its conditions, to yield the best results. Other studies have analyzed the biological effect of hydrogen peroxide on plants, for example: In amaranth seedlings, concentrations of 0.1, 1 and 5 mM H_2_O_2_ were applied to the *Amaranthus mangostanus* species without significant effects on the accumulation of betacyanin reported in comparison to the control group [28]. Hydrogen peroxide has previously been reported to be a stimulant for the production of betalains in beets (*Beta vulgaris*) [29]. In a study of peppermint plants (*Mentha piperita*), low concentrations of H_2_O_2_ (0.05, 0.10 and 0.50 mM) were applied to leaves twice during the crop cycle. The results showed no significant effect on leaf size, while an increase of between 10% and 12% was observed in the content of total phenolic compounds, determined by the Folin–Ciocalteau method with application of a concentration of 0.50 mM H_2_O_2_. No statistical difference in antioxidant capacity (ABTS and DPPH) was found (*p* > 0.05) between the control group versus the treatment groups [30].

Phenolic compounds are secondary metabolites that are involved in various plant processes, such as reproduction and growth. They are also synthesized as a defense mechanism against biotic or abiotic stress [31], so the synthesis of these metabolites can be increased using elicitors [32,33]. In one study, H_2_O_2_ concentrations of 15 and 150 mM were applied to lentil sprouts [21]. Having determined the content of total phenolic compounds using the Folin–Ciocalteau method, increases of 65% and 44% were observed with the 15 and 150 mM H_2_O_2_ treatments, respectively. The 15 mM concentration of the elicitor did not affect the yield of the lentil sprouts. The same group of researchers had previously evaluated H_2_O_2_ concentrations (20 and 200 mM) applied to lentil sprouts, reporting an increase in total antioxidant capacity two days after application [23]. It has been reported that in low concentrations, H_2_O_2_ can induce the synthesis of secondary metabolites in plants [34,35]. Therefore, the use of elicitors in plants may be a strategy for obtaining increased secondary metabolites with various potential uses. It was reported that H_2_O_2_ induces antioxidant activity and the accumulation of cardiotonic glycoside (a cardioactive secondary metabolite) in four species of the *Digitalis L* plant (*Digitalis davisiana*, *Digitalis lamarckii*, *Digitalis trojana* y *Digitalis cariensis*) [36]. In that study, plants were germinated in vitro using phytohormones, calluses were induced, and a subculture was taken. The authors used a concentration of 10 mM H_2_O_2_ for 6 h as the culture medium. Their results showed an increase in total phenolic compounds determined by the Folin–Ciocalteau method. They concluded that hydrogen peroxide treatment can be an effective method for increasing secondary metabolites. Another study [37] reported that the accumulation of capsidiol in peppers (*Capsicum annuum* L.) was induced by H_2_O_2_. The accumulation of phenolic compounds under abiotic and biotic stress has also been reported to coincide with an increase in the enzyme phenyl ammonium lyase (PAL). Plant defense and related pathways such as antioxidant enzymes, glutathione-S-transferase (GST), PAL, defense proteins, and transcription factors have been reported to be influenced by H_2_O_2_ [38,39]. It is important to emphasize that with the 125 mM H_2_O_2_ treatment, all samples presented an increase in TFC concentrations. Likewise, all the leaves at 63 days presented an increase in these secondary metabolite levels, and all the seeds presented an increase in TFC under any of the treatments. Finally, an improvement in the amounts of secondary metabolites observed with the H_2_O_2_ treatments coincides with an improvement in the antioxidant capacity measured by FRAP.

The amount or concentration of an elicitor to be used is a complex issue, and there is no universal method for determining it. There are various systems for elicitation to increase the production of secondary metabolites useful in human health, for example, in bioreactors, in vitro plant tissue cultures, or directly in calluses, sprouts, and mature plants [17,20,40]. In these systems, the quantity, concentration, exposure time and frequency of the elicitor used can vary widely. The concentration of a foliar spray for an in vivo plant tissue culture is not the same as for a mature plant because the plant cell of the former will be exposed for a longer time than the latter. In the case of foliar spray, higher concentrations can be used and should be evaluated. It should also be noted that when applied to leaves, the rate at which the hydrogen peroxide solution evaporates into the medium depends on the ambient temperature and humidity.

In our previous study [17], concentrations of 0.6, 14 and 18 mM H_2_O_2_ were tested on the same variety of amaranth (*A. hypochondriacus*), showing a moderate increase in phenolic compounds (including TPC, condensed tannins and anthocyanins). In that study, it was concluded that the concentrations of hydrogen peroxide used were sufficient to promote a certain level of stress that produces an increase in phenolic compounds. In the present study, however, we observed a stronger response, both in the concentration of phenolic compounds at different times and in different treatments and in antioxidant capacity measured by FRAP. As in the past experiment, in this study the impact (increase) observed in the amaranth seed was less noticeable than in the leaf, and no dose-dependent response was observed. It is significant to note that in the present study, the strongest and clearest responses were observed in plants treated with 125 mM H_2_O_2_, for all the variables studied, including the antioxidant capacity measured by FRAP. In the previous Espinosa-Villarreal study [17], the H_2_O_2_ treatments (0.6, 14 and 18 mM) were applied only four times every 7 days from the 40th day after germination, while in the current study the H_2_O_2_ treatments (0, 125, 250 and 400 mM) were applied 11 times every 7 days, starting at day 61 after germination. In the present study, an intermediate concentration between 250 and 400 mM (e.g., 325 mM) might perhaps have been useful to gain a more complete picture of how different concentrations of hydrogen peroxide may affect the result.

Although some studies propose the use of exogenous H_2_O_2_ application to increase the production of phenolic compounds, no study has been carried out to clarify what specific relationship exists between their expression and the elicitor.

Foliar application of H_2_O_2_ in plants can be a strategy for increasing production of phenolic compounds. However, it has been observed that the response to the application of H_2_O_2_ differs depending on the time of application, the concentration used, and the plant species. The concentration, the number of applications, the time of application, and other aspects all intervene in the cellular activities of each plant, so it is of utmost importance to determine the optimal conditions for increasing the secondary metabolites of interest for each particular system. The effectiveness of elicitation as a tool for enhancing the production of secondary metabolites depends on a complex interaction between the elicitor, the plant cell and the environment [41]. Among the main factors that may affect this interaction are the specificity of the elicitor, the concentration used and application method, the treatment intervals, the cultivation conditions, such as the growth stage, the environment and sunlight [42]. Elicitation is a tool used mainly to manipulate the metabolism to increase the presence of secondary metabolites in a food that may have health benefits. The overproduction of phenolic compounds in response to the treatments used in the study was diverse. The use of abiotic elicitors seems to be a good alternative for improving the levels of some secondary metabolites of interest and is relatively inexpensive and easy to apply, but they must be analyzed on a case-by-case basis. The main limitation of the present study is that specific compounds were not identified (using, for example, HPLC or UPLC), which would provide a better picture of which compounds increased or decreased in both leaves and seeds. However, observing a global response using colorimetric techniques represents a better measurement of the global effect of an elicitor, both in terms of the content of total phenolic compounds and in terms of antioxidant capacity.

## 4. Materials and Methods

### 4.1. Biological Reagents and Materials

Triphenyltriazine (TPTZ) 2,4,6-tris(2-pyridyl)–1,3,5-triazine ≥ 98%, ascorbic acid 99%, catechin ≥ 98%, DPPH (2,2-diphenyl-1-picrylhydrazyl) 98%, ferric chloride 97%, Folin–Ciocalteu 98% reagent, gallic acid 98%, hydrogen peroxide 3%, sodium acetate 99%, sodium carbonate 99% and vanillin 99%, from Sigma–Aldrich Corp. (St. Louis, MO, USA), and hydrochloric acid, and methanol from J.T. Baker (Center Valley, PA, USA), were used.

The seeds of *Amaranthus hypochondriacus* of the “Revancha” variety were donated by the National Institute of Forestry, Agriculture and Livestock Research (INIFAP).

### 4.2. Greenhouse Features

**Figure 1:**
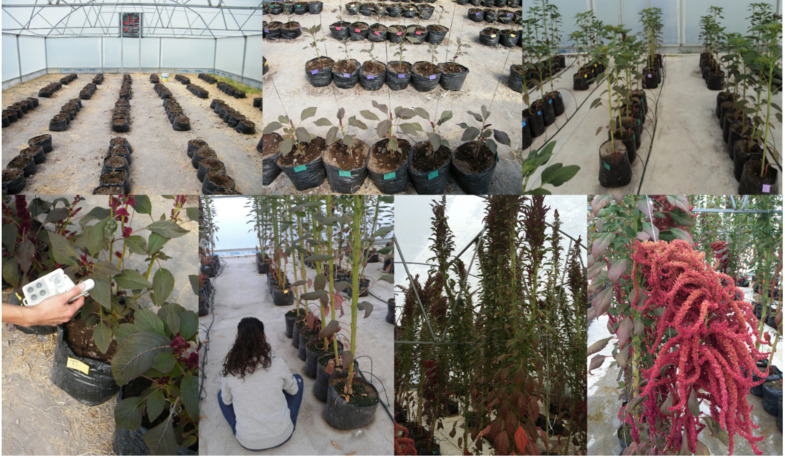
Amaranth (*Amaranthus hypochondriacus* L.) growth in the greenhouse during the experiment.

The plants were grown in a single Gothic-style greenhouse measuring 100 m^2^ and equipped with an electric ventilator (0.5 hp and 50 inches). The cladding material was a single layer of long-term polyethylene plastic. The greenhouse is oriented north-south. The study was conducted in the Engineering School at Amazcala, in the municipality of El Marqués, in the state of Queretaro, Mexico, CP. 76130 (Figure 1). This is a semi-arid region. Amazcala is located at 20°42′ 20″ north, and 100°15′ 37″ west, at 1921 m above sea level.

### 4.3. Preparations and Applications of H_2_O_2_ and Study Design

**Figure 2:**
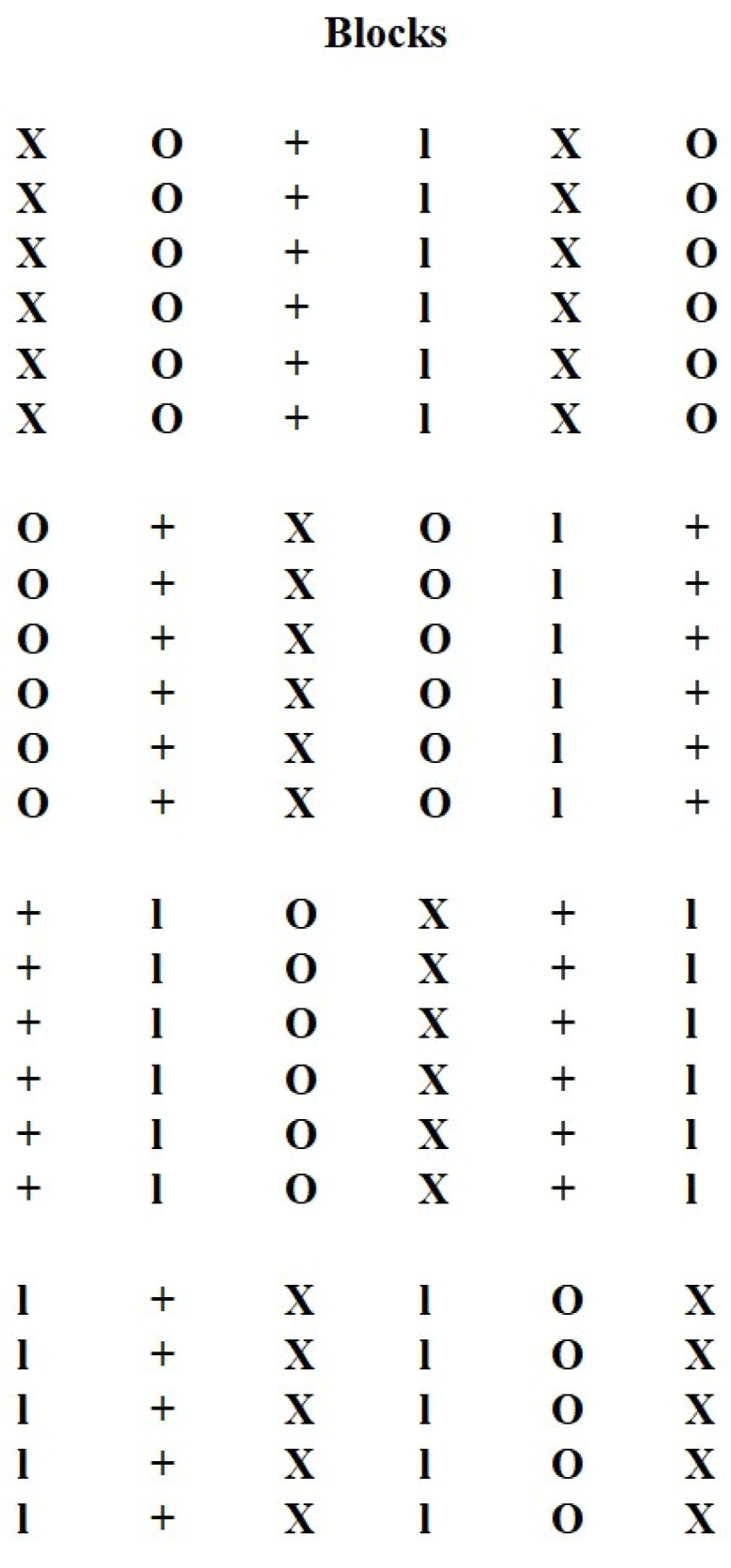
Distribution of 6 randomized blocks with 6 plants for each treatment (36 plants per treatment). Treatments: 0 (**O**), 125 (**l**), 250 (**+**) and 400 (**X**) mM hydrogen peroxide.

The concentrations of H_2_O_2_ were prepared in the plant physiology laboratory of the Amazcala Campus. De-ionized water was used for H_2_O_2_ preparations (0, 125, 250 and 400 mM). A randomized experimental study was carried out, establishing 6 random blocks with 6 plants for each treatment (36 plants) and 4 treatments (0, 125, 250 and 400 mM H_2_O_2_) (Figure 2). The cultivation began on May 1 with the transplantation of 16 cm high seedlings and the harvest of the amaranth seed was completed on September 15. The seedlings were placed in black polyethylene containers with a volume capacity of 20 L. The containers were filled with clay soil (25%), sand (25%) and *tezontle* substrate (50%). For the base nutrient solution, the universal solution developed by Steiner [43] at 40% was used. This was applied three weeks after plant germination and until the beginning of the inflorescence. The hydrogen peroxide concentrations were applied 61 days after germination and 11 times throughout the culture, every 7 days. Application was carried out by foliar spraying until the dropping point between 09:00 and 10:00 h. Using an automatic drip irrigation system, seedlings were watered for 15 min every 4 h, starting at 6:00 and ending at 18:00. When the average plant height exceeded 25 cm, the frequency was changed to every 3 h. The drip irrigation system was Eurodrip (diameter: 8000), with high turbulent flow emitters and a coefficient of variation <2%. The flow rate was 0.4 GPH (USA, Eurodrip, Guanajuato, Mexico). Each plant had its own dripper.

### 4.4. Sample Selection

During the treatment period, 3 amaranth leaf samples were taken (at 63, 90 and 127 days after sowing), and seeds were harvested on day 134. The samples were immediately dried in an oven (Shel lab 1375, Swedesboro, NJ, USA) at 40 °C until they reached a constant weight (approximately 24 h). The dry matter was ground in a mill (Thomas Scientific Model 4 Wiley, USA) with a 0.5 mm diameter sieve. The samples were stored in a deep freezer (REVCO Ultima II, Asheville, NC, USA) at −80 °C for subsequent analysis.

### 4.5. Analytical Determinations

**Proximate analysis:** Moisture, crude ash, crude fiber, crude protein and ether extract content were determined in amaranth fresh leaves and seeds following methods [44] (934.01, 923.03, 920.39, 960.52 and 920.86). To carry out each of the determinations, powdered dry matter was used. The determinations were made in triplicate.

**Determination of chlorophyll:** A SPAD 502 chlorophyll meter (Konica Minolta, Ramsey, NJ, USA) was used to determine chlorophyll. The determinations were made between 8:00 and 10:00 a.m. by means of a random sampling of the mature leaves from each plant in the study. The instrument quantifies absorbance in dimensional values from 0 to 199 in Soil Plant Analysis Development (SPAD) units [45].

### 4.6. Growth Parameters

The following growth parameters in the amaranth plants were determined throughout the crop cycle (at 41, 55, 63, 76, 90, 104 and 127 days):

**Plant height**: a 5 m (0.01 mm) flexometer FH-3m (Truper, Taiwan) was used, measuring from the surface of the substrate to the highest point of the plant above the apical meristem;

**Stem diameter**: Stem diameter was measured in millimeters using a 190 mm stainless hardened vernier (Metromex 222-A, Mexico City, MX, MEX). The measurement point was made on the main stem, 1 cm above the substrate;

**Number of leaves**: the total number of leaves of each amaranth plant was counted (36 determinations per group), 5 times during the cultivation period;

**Determination of panicle length**: panicle length (cm) was measured using a FH-3m flexometer (Truper, Jilotepec, MX, MEX) at the end of the amaranth cultivation period.

### 4.7. Antioxidant Capacity

**Determination of 1,1-diphenyl-2-picrylhydrazyl (DPPH) assay**: Antioxidant activity was determined using the free radical DPPH dissolved in methanol; in this form, the maximum absorption of the radical is at 550 nm. The DPPH was reduced by the action of the antioxidant (e.g., ascorbic acid), and the decrease in absorbance was determined. A DPPH assay was performed as described in Brand-Williams [46], with some adaptations using a microplate reader [17]. Briefly, 20 µL of the prepared sample were taken, 280 µL of 60 µM DPPH were added to it, and it was incubated for 30 min at room temperature. Subsequently, the absorbance was read at 550 nm. The antioxidant capacity value was obtained by interpolating the absorbance differential in a standard curve built with concentrations of 8.8, 17.6, 44, 88, 132 and 176 µg/mL of ascorbic acid. The values were expressed in mg of ascorbic acid equivalents (AAE)/100 g of dry matter (DM). To determine the % inhibition, 4 concentrations of extract were used: 1, 3, 5 and 10 mg/mL, and the following formula was used:

% inhibition = (A − B)/A × 100

A = Blank absorbance;

B = Sample absorbance.

To determine the IC_50_, which is the necessary amount of antioxidant sample to inhibit the radical by 50%, a linear regression was performed (of the concentrations used versus the % inhibition) to obtain the equation of the line y = mx + b, leaving IC50 = (50 − b)/m.

**Determination of ferric-reducing antioxidant power (FRAP) activity**: This method measures any solution containing antioxidants and is based on the reduction of the ferric ion complex -(2,4,6-tripyridyl-s-triazine) from (FE+3-TPTZ) to FE+2–TPTZ. The FRAP assay was performed at 37 °C and pH 3.6 following the method developed by Benzie and Strain [47]. Briefly, 60 µL of the prepared sample were taken, along with 240 µL of a solution composed of acetate buffer [10:1:1 (300 mM, pH 3.6), TPTZ (10 mM in 40 mM HCl) and ferric chloride (20 mM)]. The plate was allowed to rest for 30 min and was subsequently read at an absorbance of 593 nm. The absorbance values are compared to a standard curve made with ascorbic acid. The final results were expressed in grams of AAE per 100 g of MS. To determine the % inhibition and IC_50_, 4 concentrations were used: 2, 6, 10 and 20 mg/mL, and the same procedure as in DPPH was followed.

### 4.8. Phenol Content

**Determination of total phenolic compounds (TPC)**: The TPC of the amaranth was determined according to the Folin–Ciocalteu colorimetric method [48]. Briefly, 12 μL of the sample was plated in a 96-well ELISA plate. Quantities of 50 μL of HPLC water and 13 μL of Folin–Ciocalteu reagent are added. The sample was left to rest for 6 min. Then 125 μL of 7% sodium carbonate solution and 100 μL of HPLC water were added to adjust the volume to 300 μL. Subsequently, it was left to rest for 90 min and read at 750 nm. The concentration of total phenolic compounds was obtained by comparing with a standard curve of gallic acid, whose concentrations were: 0.02, 0.04, 0.06, 0.08, 0.10, 0.12, 0.14, 0.16, 0.18 and 0.20 mg/mL. The results were expressed in milligrams of gallic acid equivalents (GAE) per 100 g of dry matter (DM).

**Determination of condensed tannins**: The condensed tannin content was measured using the vanillin-hydrochloric acid test [49]. Briefly, 40 μL of the sample was placed in a 96-well plate. A quantity of 200 μL of freshly prepared vanillin reagent (1% vanillin in methanol and 8% HCl in methanol in a 1:1 ratio) was added. A blank was prepared with 40 μL of each concentration plus 200 μL of 4% HCl in methanol. The reactions of both samples are carried out at a temperature of 30 °C and allowed to rest for 20 min. Finally, the sample was read at an absorbance of 492 nm. The concentration of condensed tannins was calculated by comparing it with a standard catechin curve, whose concentrations were: 0.02, 0.04, 0.06, 0.08, 0.10, 0.12, 0.14, 0.16, 0.18 and 0.20 mg/mL. The results were expressed in milligrams of catechin equivalents (CE) per 100 g of DM.

**Determination of anthocyanins**: This was carried out according to the method developed by Abdel and Hucl [50]. Briefly, 0.5 g of dry sample was weighed. A quantity of 25 mL of acidified ethanol (ethanol:1N HCl in a ratio of 85:15, respectively) were added. It was vortexed at 8000 rpm for 30 min and the pH was adjusted to 1.0 with 4N HCl. The solution was centrifuged at 5000 rpm for 15 min. The supernatant was decanted into a volumetric flask and brought to 50 mL with acidified ethanol. The flask was shaken manually by vigorous inversion and the sample was read at an absorbance of 535 nm. The results were expressed in milligrams of cyanidin 3-glucoside equivalents (CGE) per 100 g of DM.

### 4.9. Statistical Analysis

The experimental data of the scale variables obtained were subjected to an analysis of variance (ANOVA) followed by a Tukey *post hoc* test to identify differences between the contrasted groups. Comparisons of the variables studied were made for different sampling times (63, 90 and 127 days) and for different treatment concentrations (0, 125, 250 and 400 mM). A 95% confidence interval and a significance level (*p* < 0.05) were used. The statistical program SPSS V23 for Windows was used.

## 5. Conclusions

Foliar applications of H_2_O_2_ (125, 250 and 400 mM) 11 times throughout the amaranth growing cycle had positive results, moderately increasing the antioxidant capacity, total phenolic compounds, condensed tannins, and anthocyanins in amaranth leaf at different sampling times and in different treatments without detriment to growth parameters, chlorophyll content, or proximal chemical composition. The increase was less pronounced in amaranth seed than in the leaf. These increases in leaves and seeds were observed with the application of different concentrations at different times, although no dose-response effect was observed in any of the variables analyzed. In this study, the concentration of 125 mM H_2_O_2_ proved to produce the most favorable and strongest response. Further study is necessary in this regard.

## Figures and Tables

**Table 1:** Results of the proximate chemical analysis in amaranth leaf and seed (*Amaranthus hypochondriacus* L.).

Sample (Time)	H_2_O_2_ Treatments (mM)	Moisture (%)	Crude Ash ^a^ (%)	Crude Fiber ^a^ (%)	Crude Protein ^b^ (%)	Ether Extract ^a^ (%)
Leaf day 63	0	76.30 ± 2.1	3.66 ± 0.1	2.62 ± 0.1	3.93 ± 0.1	0.29 ± 0.1
	125	78.00 ± 1.7	3.76 ± 0.2	2.64 ± 0.2	3.87 ± 0.2	0.41 ± 0.2
	250	77.89 ± 2.0	3.84 ± 0.1	2.63 ± 0.1	3.98 ± 0.2	0.37 ± 0.2
	400	76.40 ± 1.5	3.78 ± 0.1	2.70 ± 0.1	3.94 ± 0.2	0.45 ± 0.2
Leaf day 90	0	76.58 ± 1.0	3.73 ± 0.1	2.64 ± 0.2	3.94 ± 0.2	0.30 ± 0.2
	125	75.76 ± 2.0	3.66 ± 0.2	2.59 ± 0.1	3.91 ± 0.2	0.34 ± 0.2
	250	76.53 ± 2.1	3.67 ± 0.1	2.65 ± 0.2	3.97 ± 0.2	0.42 ± 0.1
	400	76.65 ± 1.5	3.68 ± 0.1	2.60 ± 0.1	3.90 ± 0.2	0.38 ± 0.2
Leaf day 127	0	76.74 ± 2.6	3.61 ± 0.1	2.58 ± 0.1	3.96 ± 0.1	0.35 ± 0.1
	125	76.66 ± 2.0	3.70 ± 0.1	2.57 ± 0.1	3.90 ± 0.2	0.26 ± 0.1
	250	77.46 ± 1.5	3.77 ± 0.1	2.72 ± 0.1	3.98 ± 0.1	0.52 ± 0.1
	400	76.90 ± 1.0	3.73 ± 0.2	2.66 ± 0.1	3.90 ± 0.2	0.38 ± 0.2
Seed	0	9.08 ± 1.2	3.12 ± 0.1	5.90 ± 0.2	17.08 ± 0.1	4.30 ± 0.2
	125	9.72 ± 1.0	3.01 ± 0.1	5.95 ± 0.1	17.18 ± 0.2	4.32 ± 0.1
	250	10.32 ± 1.0	3.02 ± 0.1	5.95 ± 0.1	17.11 ± 0.1	4.22 ± 0.1
	400	10.26 ± 1.5	2.96 ± 0.1	5.99 ± 0.2	17.12 ± 0.1	4.24 ± 0.2

Results are shown as the average of three determinations ± 1 standard deviation. No statistical differences (*p* > 0.05) were observed between the treatments; ^a^ (% dry matter); ^b^ (% dry matter N × 6.25).

**Table 2:** Results of the growth parameters of amaranth (*Amaranthus hypochondriacus* L.).

H_2_O_2_ Treatments (mM)	41 Days	55 Days	63 Days	76 Days	90 Days	104 Days	127 Days
Plant height ^a^ (cm)							
0	44.94 ± 9.4	82.47 ± 17.3	121.80 ± 17.6	164.91 ± 22.6	218.0 ± 32.6	251.61 ± 35.0	297.0 ± 45.7
125	45.19 ± 11.2	77.25 ± 13.5	116.14 ± 15.8	158.75 ± 19.7	208.63 ± 23.2	254.25 ± 35.2	295.19 ± 42.0
250	46.85 ± 8.4	82.64 ± 15.5	122.05 ± 20.5	166.11 ± 27.1	213.50 ± 27.1	267.33 ± 36.5	309.88 ± 45.2
400	43.86 ± 9.0	79.61 ± 17.0	120.11 ± 18.4	164.62 ± 23.7	211.14 ± 26.0	274.02 ± 44.0	315.37 ± 47.0
Stem diameter ^a^ (cm)							
0	1.04 ± 0.1	1.60 ± 0.1	1.83 ± 0.2	2.01 ± 0.3	2.22 ± 0.3	2.40 ± 0.3	2.50 ± 0.4
125	1.03 ± 0.1	1.61 ± 0.1	1.82 ± 0.2	2.00 ± 0.3	2.13 ± 0.3	2.33 ± 0.4	2.43 ± 0.4
250	1.05 ± 0.1	1.64 ± 0.1	1.82 ± 0.2	2.00 ± 0.2	2.20 ± 0.3	2.34 ± 0.3	2.45 ± 0.3
400	1.01 ± 0.1	1.61 ± 0.1	1.86 ± 0.2	2.00 ± 0.2	2.14 ± 0.2	2.31 ± 0.3	2.45 ± 0.3
Number of leaves							
0	22.83 ± 3.4	67.42 ± 22.0	89.11 ± 23.0	153.42 ± 46.3			
125	23.11 ± 5.1	67.53 ± 18.0	90.00 ± 23.0	156.13 ± 26.6			
250	24.40 ± 4.8	61.00 ± 14.0	87.00 ± 24.6	153.24 ± 38.0			
400	21.50 ± 4.0	57.00 ± 17.1	83.30 ± 24.5	157.00 ± 37.3			
Panicle length ^a^ (cm)							
0							102.00 ± 20.0
125							96.22 ± 17.4
250							94.44 ± 24.4
400							102.00 ± 20.2

^a^ Results are expressed as the average of 36 determinations ± standard deviation. No statistical difference was found (*p* > 0.05) in any of the studied variables. The different treatments are compared in each of the columns.

**Table 3:** Results of the antioxidant capacity by DPPH and FRAP methods in amaranth leaf and seed (*Amaranthus hypochondriacus* L.).

Treatments	Leaf (63 Days)			Leaf (90 Days)			Leaf (127 Days)			Seed		
H_2_O_2_ (mM)	mg AAE *	(% Inh) ^x^	IC_50_^y^	mg AAE *	(% Inh) ^x^	IC_50_^y^	mg AAE *	(% Inh) ^x^	IC_50_^y^	mg AAE *	(% inh) ^x^	IC_50_^y^
	DPPH, expressed in mg of ascorbic acid equivalents/100 g dry matter											
0	472.5 ± 1.5 ^a^	78.3 ± 1 ^a^	36	288.0 ± 0.8 ^a^	58.8 ± 1 ^a^	62.6	585.0 ± 3.3 ^a^	90.2 ± 1 ^a^	19.9	25.0 ± 0.8 ^a^	17.6 ± 1 ^a^	102.5
125	556.4 ± 0.3 ^b^	87.2 ± 1 ^b^	24	251.8 ± 0.4 ^b^	55.0 ± 1 ^b^	67.8	510.4 ± 1.0 ^b^	82.3 ± 2 ^b^	30.6	16.7 ± 0.8 ^b^	16.8 ± 0 ^b^	101.7
250	493.8 ± 3.2 ^c^	80.6 ± 0 ^a^	33	118.9 ± 1.1 ^c^	41.0 ± 0 ^c^	86.9	511.9 ± 0.7 ^b^	82.5 ± 1 ^b^	30.4	32.4 ± 2.4 ^c^	18.4 ± 1 ^a^	99.3
400	392.2 ± 1.0 ^d^	69.9 ± 1 ^c^	47.6	124.9 ± 0.6 ^d^	41.6 ± 1 ^c^	86.0	513.2 ± 4.5 ^b^	82.6 ± 1 ^b^	30.2	26.8 ± 0.4 ^a^	17.8 ± 0 ^a^	100.1
	FRAP, expressed in g of ascorbic acid equivalents/100 g dry matter											
0	2.66 ± 0.0 ^a^	78.8 ± 1 ^a^	95.7	6.00 ± 0.0 ^a^	86.0 ± 2 ^a^	50.1	7.00 ± 0.0 ^a^	88.1 ± 1 ^a^	36.5	0.66 ± 0.0 ^a^	24.6 ± 0 ^a^	123.0
125	3.26 ± 0.0 ^b^	80.1 ± 0 ^a^	87.5	6.54 ± 0.0 ^b^	87.1 ± 1 ^a^	42.8	8.57 ± 0.0 ^b^	91.5 ± 1 ^b^	15.1	0.66 ± 0.0 ^a^	24.6 ± 0 ^a^	122.9
250	2.64 ± 0.0 ^a^	78.8 ± 1 ^a^	95.9	5.65 ± 0.0 ^c^	85.2 ± 2 ^a^	54.9	7.10 ± 0.0 ^c^	88.3 ± 2 ^a^	35.1	0.63 ± 0.0 ^a^	24.5 ± 0 ^a^	123.4
400	2.75 ± 0.0 ^c^	79.0 ± 2 ^a^	94.4	5.02 ± 0.0 ^d^	83.9 ± 2 ^b^	63.5	7.68 ± 0.0^d^	89.6 ± 1^a^	27.2	0.59 ± 0.0^b^	24.4 ± 1^a^	123.9

The results are shown as the average of four determinations ± one standard deviation. Different letters indicate a statistical difference (*p* < 0.05) comparing different H_2_O_2_ treatments (0 vs. 125 vs. 250 vs. 400 mM) in the same column. * AAC = ascorbic acid equivalents. ^x^ = % inhibition. ^y^ = IC_50_ (mg/mL).

**Table 4:** Results of the content of total phenolic compounds (TFC) in amaranth leaf and seed (*Amaranthus hypochondriacus* L.).

H_2_O_2_ Treatments (mM)							
Sample (Days)	0	125		250		400	
			C (%) *		C (%) *		C (%) *
TFC expressed in mg of gallic acid equivalents/100 g dry matter.							
Leaf (63 days)	254.40 ± 2.2 ^a^	274.17 ± 4.2 ^b^	8	285.91 ± 2.0 ^c^	12	317.48 ± 0.6 ^d^	25
Leaf (90 days)	373.66 ± 2.9 ^a^	453.84 ± 2.2 ^b^	21	486.99 ± 5.0 ^c^	30	362.01 ± 3.3 ^d^	−3
Leaf (127 days)	486.41 ± 4.0 ^a^	586.60 ± 1.6 ^b^	21	438.90 ± 3.4 ^c^	−10	435.24 ± 0.0 ^c^	−11
Seed	130.51 ± 1.7 ^a^	162.47 ± 1.7 ^b^	24	187.92 ± 1.6 ^c^	44	145.90 ± 1.9 ^d^	12

The results are shown as the average of four determinations ± one standard deviation. Different letters indicate a statistical difference (*p* < 0.05) comparing different H_2_O_2_ treatments (0 vs. 125 vs. 250 vs. 400 mM) in the same row. * Percentage change compared to the control group.

**Table 5:** Content of condensed tannins in amaranth leaf and seed (*Amaranthus hypochondriacus* L.).

H_2_O_2_ Treatments (mM)							
Sample (Days)	0	125		250		400	
			C (%) *		C (%) *		C (%) *
Condensed tannins expressed in mg of catechin equivalents/100 g dry matter.							
Leaf (63 days)	948.65 ± 25.0 ^a^	1045.72 ± 15.7 ^b^	10	1333.30 ± 9.6 ^c^	41	924.69 ± 7.4 ^a^	−3
Leaf (90 days)	1632.28 ± 8.4 ^a^	2225.76 ± 46.6 ^b^	36	1495.61 ± 22.5 ^c^	−8	1244.73 ± 14.1 ^d^	−24
Leaf (127 days)	1274.26 ± 8.5 ^a^	1475.17 ± 16.8 ^b^	16	1837.12 ± 12.0 ^c^	44	1518.0 ± 12.0 ^d^	19
Seed	231.75 ± 6.2 ^a^	237.27 ± 5.1 ^a^	2	243.75 ± 19.7 ^a^	5	222.55 ± 11.3 ^a^	−4

The results are shown as the average of four determinations ± one standard deviation. Different letters indicate a statistical difference (*p* < 0.05) comparing different H_2_O_2_ treatments (0 vs. 125 vs. 250 vs. 400 mM) in the same row. * Percentage change compared to the control group.

**Table 6:** Anthocyanin content in amaranth leaf and seed (*Amaranthus hypochondriacus* L.).

H_2_O_2_ Treatments (mM)							
Sample (Days)	0	125		250		400	
			C (%) *		C (%) *		C (%) *
Anthocyanin expressed in mg of cyanidin-3-glucoside equivalents/100 g dry matter.							
Leaf (63 days)	667.54 ± 0.6 ^a^	875.23 ± 0.6 ^b^	31	840.41 ± 0.5 ^c^	26	814.01 ± 1.1 ^d^	22
Leaf (90 days)	870.13 ± 1.0 ^a^	926.40 ± 1.0 ^b^	6	887.38 ± 1.8 ^c^	2	907.52 ± 0.2 ^d^	4
Leaf (127 days)	832.0 ± 0.6 ^a^	893.07 ± 0.2 ^b^	7	849.50 ± 0.9 ^c^	2	850.75 ± 0.7 ^c^	2
Seed	130.65 ± 0.2 ^a^	127.10 ± 0.6 ^a^	−3	132.66 ± 0.6 ^a^	2	167.37 ± 1.5 ^c^	28

The results are shown as the average of four determinations ± one standard deviation. Different letters indicate a statistical difference (*p* < 0.05) comparing different H_2_O_2_ treatments (0 vs. 125 vs. 250 vs. 400 mM) in the same row. * Percentage change compared to the control group.

## Data Availability

Not applicable.
